# The association between car accident fatalities and children’s fears: A study in seven EU countries

**DOI:** 10.1371/journal.pone.0181619

**Published:** 2017-08-03

**Authors:** Viviane Kovess-Masfety, David Sowa, Katherine Keyes, Mathilde Husky, Christophe Fermanian, Adina Bitfoi, Mauro Giovanni Carta, Ceren Koç, Dietmar Goelitz, Sigita Lesinskiene, Zlatka Mihova, Roy Otten, Ondine Pez

**Affiliations:** 1 EHESP Rennes, Sorbonne Paris Cité, Paris, France; 2 EA 4057 Université Paris Descartes, Paris, France; 3 Mailman School of Public Health, Columbia University, New York, New York, United States of America; 4 Université de Bordeaux EA4139, Institut Universitaire de France, Bordeaux, France; 5 The Romanian League for Mental Health, Bucharest, Romania; 6 Centro di Psichiatria di Consulenza e PsicosomaticaAziendaOspedalieroUniversitaria di Cagliari, Italy; 7 Yeniden Health and Education Society, Istanbul, Turkey; 8 Friedrich-Alexander University Erlangen-Nurenberg, Erlangen, Germany; 9 Clinic of Psychiatry, School of Medicine, University of Vilnius, Vilnius, Lithuania; 10 New Bulgarian University, Sophia, Bulgaria; 11 Behavioural Science Institute, Radboud University Nijmegen, Pluryn, Research & Development, Nijmegen, The Netherlands; Stellenbosch University, SOUTH AFRICA

## Abstract

Children’s fear of a car accident occurring to parents or themselves has been used as a concrete example to illustrate one of the symptoms of anxiety disorders such as separation anxiety and generalized anxiety. However, its usage across countries may be questionable where the prevalence of this specific type of injury differs. This cross-sectional study compares samples from seven diverse European countries (Bulgaria, Germany, Italy, Lithuania, Netherlands, Romania, Turkey) to see if an environmental exposure, car accident death rate per 100,000 people (country-wide from WHO data), is associated with children’s self-report of car accident fears. In this study, 6–11 year-old children were surveyed by a diagnostic instrument (Dominic Interactive) about several situations and asked if they believed they were similar to a fictional child depicted in said situations. Mothers were surveyed for additional sociodemographic information. Multivariable logistic regression was used to adjust for covariates including mother’s age, mother’s education, single parenting, and mother’s professional inactivity. We report a monotonic relationship between higher car accident death rates and the prevalence of children reporting fear of parent’s or own accident. Relative to a reference of 3.9 deaths per 100,000 people, children’s odds of reporting fear of parent’s accident ranged from 1.99 (95% CI 1.51–2.61) times to 4.84 (95% CI 3.68–6.37) times as the risk of death by car accident increased across countries. A similar result arose from fear of child’s own accident, with significant ORs ranging from 1.91 (95% CI 1.53–2.40) to 2.68 (95% CI 2.07–3.47) alongside increased death rates. Given that reporting of these fears accompanies correspondingly high accident death rates, the pertinence of using fear of car accidents as an illustration for some diagnostic item for mental disorders cross-nationally appears to be an issue.

## Introduction

Road accidents exact a heavy human and economic toll, accounting for over a million worldwide deaths per year and injuring many more, making such events a major public health issue. According to the World Health Organization [WHO] Global Status Report on Road Safety [1], road traffic injuries are currently the 8^th^ leading cause of death globally and, if current trends continue unabated, are projected to be the 5^th^ leading cause by 2030. Rates vary by country, but middle income and developing countries typically suffer the greatest burden as their motorization increases and often outpaces infrastructural development [2–4]. The repercussions are not only physical and financial; car accidents exact a psychological toll on survivors, often resulting in post-traumatic stress disorder and anxiety [5]. In addition, evidence suggests that even persons not directly involved in traumatic experiences can experience lasting psychological effects and fear acquisition due to their vicarious perception of said events [6–8]. In fact, Ollendick and King [9] found that some children report high levels of fear from events, including car accidents, mainly from negative information from their parents.

Fears in children and adolescents have been measured in quite diverse countries and cultures; they have been considered as a normal part of development and reported to change with cognitive and social experiences. Fears usually decrease as children get older with some exceptions such as fear of criticism, school related and peer related fears. Overall, girls report more fears than boys [10]. Cross cultural differences have been examined in many contexts. A past study by Ollendick *et al*. [11] found children’s fears to be invariant between relatively similar countries (namely the United States, Australia, and Britain), with 9 of 10 most commonly reported fears being the same in all three countries and having similar prevalence. Other studies have reported that childhood fears were, at least to some extent, culturally determined; Bedouins report more fear than Israelis [12], for example, and black South Africans children report more fears than white children[13]; these findings have been replicated in a US study comparing African American, Hispanic to white children yielding significant effects of ethnicity[14]. In addition the course of fear expression seems not to be universal: in some countries the age trends are different than in others [15].

Most of these comparisons have used some versions of the Fear Survey Schedule for Children which contained questions on the fear of being hit by a car and a question on the fear of having someone in the family get in an car accident. Both questions are part of factor 1 type of fear labeled “Death and Danger” which is found consistent across countries and cultures as when comparing Trinidadian children to US children; however, in this study the factors were heterogeneous across age, sex and nationality[16]. The authors hypothesized that these differences may be due to linguistic equivalence and functional equivalence which related to the meaning of the experiences across cultures. For example, in the Turkish version “fear of god” had to be deleted since it was widely endorsed and considered as a positive feeling [17]. Furthermore, they suggested that the items contained into the scales may have been experienced in different frequencies of either direct or indirect exposures to the items among these common fears was that of being injured by an automobile. Yet, the researchers pointed out that the extent of children’s fear of these stimuli could be due to two mechanisms: direct exposure to stressful events leading to rational fear of said events, or a disproportionate, disordered response to a hypothetical situation. [11]

Furthermore, a study compared fears of 5 to 6 year old children across time between Estonia, a former socialist country, and Finland, who joined EU much sooner, considered fear as an indicator of societal well-being. The authors theorized that it was important to ask the children themselves about their feelings and adapted the fear questionnaire into a version that used pictures and situations happening to a fictive child to be endorsed. They found that prevalence of self-reported fears increased over the ten year period especially among the Estonian children and that despite an increase of welfare, safety decreased as insecurity increased. Some fears seems to be universal whether some other are very much context dependent including exposure to media. Finally the family composition and some socio economic factors influence fears, as assessed both qualitatively as quantitatively [18]. The role of socio demographic and socio economic factors on children fears was also established in a child British survey as low social class children have a greater propensity to some fears than high social class children [19] as it was on a Turkish survey where education level and family income was related to overall fear level[17]

The relationship between children’s fears and anxiety symptoms has been a target of research because the stimuli that elicit these responses may vary between countries; therefore, presence of certain fears may differ in diagnostic value depending on context[11]. Anxiety disorders are believed to be common in preadolescent children, though actual prevalence is debated and has been reported to be present in about 2–40% of the population [20] and to vary between countries [21]. Yet, a generalizable and cross-cultural exportable method of validly diagnosing said disorders presents a considerable challenge [22]. Since not all children experience the same exposure opportunity to traumatic events and ensuing disorders (here, knowledge of automobile risks and ensuing fear) [23], this questioned the value of comparisons between groups where some diagnoses will be drawn from answers to questions on events that not all study participants were at identical risk of exposure or subject to the same environmental pressures (say, number of automobiles).

To address this question, the current study uses data collected from the School Children Mental Health Evaluation project (SCHME), which aimed to assess school children’s mental health in the European region via a large multisite school-based survey among school children aged 6–11 in seven EU countries (Bulgaria, Germany, Italy, Lithuania, the Netherlands, Romania, and Turkey) in 2010 [24]. Among the child mental health information questions were asked to the children whether they fear their parents or themselves to be victims of a car accident, which will be compared to the car accident rates in their country of appurtenance.

The diversity of countries allows a large range of situations, and based on prior literature we hypothesized that 1) the rates of fears concerning car accident will be largely different across countries with variable cultural histories and levels of economic development; 2) these fears will be independently correlated to some socio demographics belonging to the children including gender and age as to some parental characteristics such as mother education, family situation, age and activity status; and 3) that controlling for all these factors, car accident rates as reported in the diverse countries will remain a significant correlate for children fears of care accident for their parents and themselves.

## Materials and methods

### Sample characteristics

The School Children Mental Health Europe (SCMHE) study is a cross-sectional survey of European school children aged 6 to 11. The sample included data collected in 2010 in Germany, Italy, the Netherlands, Lithuania, Romania, Bulgaria, and Turkey. Country-specific sampling procedures have been described elsewhere in detail [24]. First, approximately 45–50 schools were approached per country (a greater number of schools were approached in Germany and the Netherlands). Second, 48 children were then randomly selected in each school, except in the Netherlands, where a lesser number of schools participated. Children absent on the day of the survey were excluded. Among participating schools, between 50.5% (Turkey) and 90.5% (Netherlands) selected children participated. Within each country, except for Italy where it was not possible, data were weighted to adjust the probability of being selected considering the size of the school.

Ultimately, for 8,439 participants data from at least one informant were available (n = 8,120 for child self-reports and n = 6,031 for parent questionnaires).

#### Ethics statement

Parents received an information letter and a consent form to be signed and returned to the school if they did not want their child to participate. This procedure was approved by each country’s ethic committee. All participating countries had the support of their government, including their ministers of education and health and received ethical approval from relevant authorities. Specific procedures were used in Germany and Turkey where such committees operate differently. In addition each country provided authorizations from school authorities. In Bulgaria: The Deputy Minister of Education, Youth and Science of the Republic of Bulgaria; in Germany approval was obtained through landers: a) Ministry of Education, Science and Culture, Mecklenburg-Vorpommern b) State school authority, Luneburg c) Ministry of Education and Culture of Schleswig-Holstein country; in Lithuania: the Ministry of Education and Science of the Republic of Lithuania; in the Netherlands: the Commission of Faculty Ethical Behavior Research; in Romania the Bucharest School Inspectorate General Municipal, in Turkey: the Istanbul—directorate of National Education; and in Italy: the ethical committee of the Association of European Mediterranean University. Data were anonymized for analysis.

### Instruments

#### Dominic interactive

A computer-assisted diagnostic tool called Dominic Interactive (DI) was used to allow children to self-report their own mental health symptoms, ultimately providing a DSM-IV-based likelihood assessment for seven common mental health conditions [25, 26]. The program presents children with 91 situations in which a character, Dominic, is exposed to a variety of situations and asks yes or no questions as to whether children are like Dominic. Questions are accompanied by cartoon drawings, text, and verbal cues. Characters are customizable by gender and race and the text and audio is changed to suit the particular country.

Questionnaires were administered to children under the supervision of a research assistant with a neutral attitude to ensure they remained focused. Generally, children took 10–15 minutes to complete all 91 questions, without the option to skip questions.

**Fig 1:**
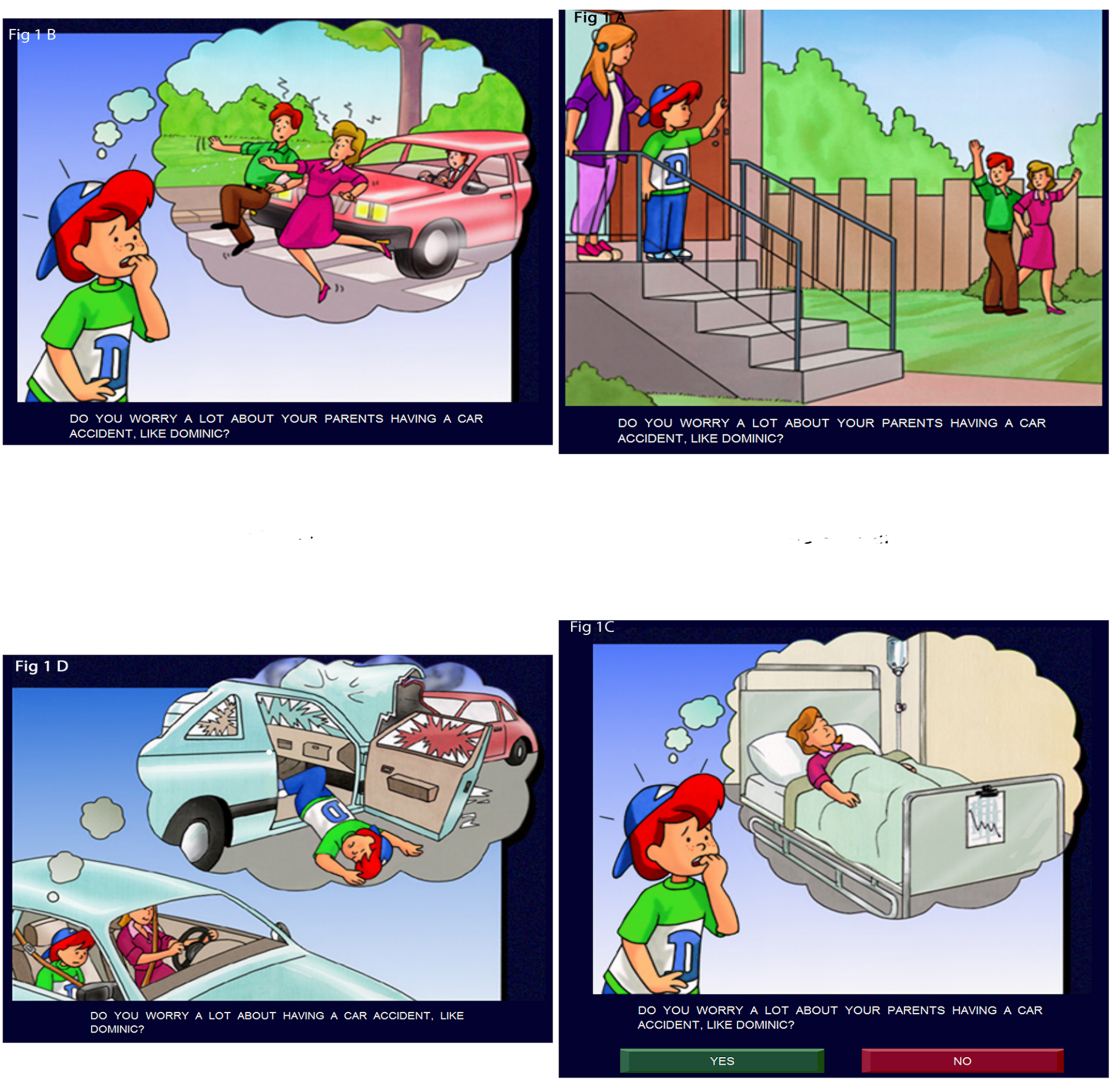
Pictures and text illustrating DI survey questions. (A-C) accompany, “*Do you worry a lot about your parents having a car accident*, *like Dominic*?”, whereas (D) accompanies the question, “*Do you worry a lot about having a car accident*, *like Dominic*?*”*. Reprinted from the Dominic Interactive under a CC BY license, with permission from DIMAT.

The DI uses DSM-IV criteria to provide a likelihood assessment for seven diagnoses: specific phobias, depression/dysthymia, oppositional defiance, conduct problems, ADHD, separation anxiety disorder (SAD), and generalized anxiety disorder (GAD). For each of the DSM IV criteria the author had to find concrete examples to make draws enabling the kids to figure out a concrete example of a rather complex symptom. For separation anxiety one the criteria “persistent and excessive worry about losing, or about possible harm befalling, major attachment figures” has been illustrated by a car accident happening to his parents as one of the frequent and easily understandable event that a child could worry about. Similarly for illustrating one of the criteria for Generalized Anxiety: “Children may worry excessively about personal safety” The author has designed a draw of a child victim of a car accident (Fig 1). An affirmative “yes” answer was the outcome of interest.

The DI has shown satisfactory internal consistency [27] as well as cross-country applicability [28]. Using the SCMHE sample, reliability values for scale scores were good to high in every country, and the factor structure was confirmed for all countries. However a thorough examination of measurement invariance provided evidence for cross-country test score comparability of 5 of the 7 scales and partial scale score invariance of the 2 anxiety scales that contain the two questions referring to car accidents.

Additional information on children’s and parent’s sociodemographic was collected from parent in a self-administered questionnaire sent back in a sealed envelope to the school except in the Netherlands where this was completed trough a dedicated website.

#### Motor vehicle-associated variables

Though the SCHME data included information on the environment of each child, these data were deemed too subjective. Thus, motor vehicle and transportation information was collected from outside internationally recognized sources, including the European Union Road Federation and the World Health Organization. Variables were created from these data and considered for greatest validity, with the final variable to be included in multivariable logistic regression. Ultimately, “2010 car accident death rate per 100,000 people” from the WHO Global Status Report on Road Safety [1] was selected for final analysis as a categorical exposure with the lowest value, 3.9 per 100,000 (from the Netherlands), chosen as the reference.

#### Variable selection and statistical analysis

In addition to the main exposure, several social possible confounders were considered. During initial univariate logistic regression analyses, all variables that were associated with p≤0.2 were selected for further analysis. After all variables were combined in a multivariable logistic regression, covariates with p≥0.05 were removed in a backwards stepwise fashion. Variables lost at this step from the child’s fear of parental accident model included mother’s age, single child status, maternal professional inactivity and child’s injury in the past year. For the child’s fear of their own accident model, variables excluded at this step were maternal education.

We included fixed effects of country to account for potential within-country correlation. Odds ratios were the measure of effect due to the data’s cross-sectional nature. All calculations were performed in SAS 9.4.

## Results

Demographic characteristics are summarized in Table 1. Child’s age, maternal age, maternal education, single parent households, and mother’s professional inactivity were all found to be unequally distributed among the study nations. Distribution of children’s gender, however, was found not to vary (p = 0.58). Additionally, affirmative responses to both parental and child car accident questions were also found to vary between nations (p<0.0001). Italy exhibited the lowest prevalence of fear of parent’s accident, with 27.2%, while Lithuania had the highest (79.3%). In the case of child’s own accident, Italy again was lowest (40.4%) and Lithuania highest (76.5%), but these values were not notably far from the next most extreme values (Netherlands with 42.8%, Turkey with 70.3%). The majority of sociodemographic variables found to be associated with fear responses in univariate models, including single child status, number of children, and parent’s perception of family burden due to the child, dropped out during multivariable analysis. Children’s self-reported fears of parent’s (p<0.0001) or their own accident (p<0.0001) were found to significantly vary by country.

**Table 1:** Distribution of demographic variables in study nations.

	Bulgaria	Germany	Italy	Lithuania	Netherlands	Romania	Turkey	
	% (n) n = 1385	% (n) n = 844	% (n) n = 757	% (n) n = 1278	% (n) n = 1657	% (n) n = 1397	% (n) n = 921	T = 8,239 p-value*
**Gender**								
Male	51.7 (716)	51.9 (438)	48.6 (368)	51.4 (657)	52.2 (865)	52.0 (727)	49.3 (454)	0.58
Female	48.3 (669)	48.1 (406)	51.4 (389)	48.6 (621)	47.8 (792)	48.0 (670)	50.7 (467)	
**Child’s age**	8.8 (1382)	8.5 (889)	8.2 (757)	8.9 (1114)	9.0 (1660)	8.7 (1384)	8.7 (797)	<0.0001
**Maternal age**								
Under 35	58.2 (626)	26.5 (125)	11.8 (89)	51.4 (567)	13.7 (98)	55.3 (640)	44.8 (281)	<0.0001
35–40 years	28.8 (309)	38.9 (183)	32.5 (246)	27.7 (306)	33.4 (238)	27.6 (319)	33.4 (210)	
Over 40 years	13.0 (140)	34.6 (163)	55.7 (421)	20.9 (231)	52.9 (377)	17.1 (198)	21.8 (137)	
**Maternal education**								
High school and above	43.0 (420)	56.1 (230)	76.2 (349)	58.2 (578)	69.8 (429)	32.4 (312)	19.6 (127)	<0.0001
High school only	47.6 (465)	38.1 (156)	22.05 (101)	30.6 (304)	29.3 (180)	50.0 (481)	26.7 (173)	
High school not completed	9.4 (92)	5.9 (24)	1.75 (8)	11.3 (112)	0.98 (6)	17.6 (169)	53.8 (349)	
**Single parent**	15.4 (164)	22.9 (102)	7.4 (53)	25.5 (293)	7.5 (50)	12.4 (146)	8.2 (55)	<0.0001
**Mother professionally inactive**	23.5 (220)	22.5 (87)	21.3 (152)	40.8 (425)	20.1 (121)	32.9 (336)	69.3 (442)	<0.0001
**Fear parent’s accident**	69.3 (960)	54.0 (483)	27.2 (206)	79.3 (1014)	39.1 (1503)	74.7 (1044)	76.6 (705)	<0.0001
**Fear own accident**	61.9 (857)	46.0 (411)	40.4 (306)	76.5 (977)	42.8 (643)	59.4 (830)	70.3 (647)	<0.0001

**Note.** Confirmation of child’s fear of parent’s or their own car accident were both found to be statistically related to country.

*Chi-square test for independent outcomes between countries, except child’s age (ANOVA).

In 2010 the car accident death rates per 100,000 people reported by the WHO Global Status Report on Road Safety were respectively: 3.9 for the Netherlands, 4.7 for Germany, 7.2 for Italy, 10.4 for Bulgaria, 11.1 for Lithuania and Romania and 12.0 for Turkey.

Two series of analyses are presented in Tables 2 and 3 in which the models using those rates were associated with child’s fear of parental car accident, first adjusted by parent and children-reported variables and second by child-reported variables only (the higher number of participants is noted in the second model, as child data were parent’s information was missing was also included).

**Table 2:** Association between demographic factors and car accident death rate with fear of parental car accident across seven countries in Europe.

Determinant	All variable AOR (n = 4800)	p-value	Child variable AOR (n = 7755)	p-value
Child’s age	0.91 (0.87, 0.96)	<0.01	0.91(0.88, 0.94)	<0.01
Female gender	1.35 (1.19, 1.54)	<0.01	1.38(1.25, 1.52)	<0.01
Maternal education	—	0.02		
High school and above	Reference			
High school only	1.20 (1.04, 1.38)	0.01		
High school not completed	1.23 (0.99, 1.54)	0.06		
Single parent	1.26 (1.04, 1.52)	0.02		
Car accident death rate (per 100,000)	—	<0.01	—	<0.01
3.9 (Netherlands)	Reference		Reference	
4.7 (Germany)	1.99 (1.51, 2.61)	<0.01	1.76(1.49, 2.10)	<0.01
7.2 (Italy)	0.65 (0.50, 0.84)	<0.01	0.53(0.44, 0.65)	<0.01
10.4 (Bulgaria)	3.64 (2.92, 4.55)	<0.01	3.48(2.98, 4.06)	<0.01
11.1 (Lithuania, Romania)	5.24 (4.28, 6.43)	<0.01	5.16(4.48, 5.94)	<0.01
12.0 (Turkey)	4.84(3.68, 6.37	<0.01	4.94(4.07, 6.00)	<0.01

Note. Relevant covariates associated with child’s fear of parent’s car accident. AOR: Adjusted odds ratios presented with 95% confidence intervals, “Child variable AOR” presents association adjusted by child demographic variables, and “all variable AOR” column presents association adjusted by both child- and parent-reported variables. All variables analyzed using multivariable logistic regression.

**Table 3:** Association between demographic factors and car accident death rate with fear of child’s own car accident across seven countries in Europe.

Determinant	All variable AOR (n = 5000)	p-value	Child variable AOR (n = 7755)	p-value
Child’s age	0.86 (0.82,0.89)	<0.01	0.84 (0.82, 0.87)	<0.01
Female gender	1.56 (1.39, 1.76)	<0.01	1.58 (1.44, 1.73)	<0.01
Maternal age	—	0.01		
Under 35 years	Reference			
35–40 years	0.90 (0.78, 1.04)	0.15		
Over 40 years	0.79 (0.67, 0.93)	<0.01		
Single parent	1.24 (1.04, 1.48)	0.02		
Mother professional inactivity (“seeking job” marked inactive)	1.22 (1.07, 1.39)	<0.01		
Car accident death rate (per 100,000)	—	<0.01	—	<0.01
3.9 (Netherlands)	Reference		Reference	
4.7 (Germany)	1.01 (0.76, 1.34)	0.95	1.06 (0.89, 1.26)	0.49
7.2 (Italy)	0.86 (0.68, 1.08)	0.19	0.78 (0.65, 0.93)	0.01
10.4 (Bulgaria)	1.91 (1.53, 2.40)	<0.01	2.12 (1.82, 2.47)	<0.01
11.1 (Lithuania, Romania)	2.55 (2.08, 3.13)	<0.01	2.70 (2.36, 3.09)	<0.01
12.0 (Turkey)	2.68 (2.07, 3.47)	<0.01	3.01 (2.50, 3.62)	<0.01

Note. Relevant covariates associated with child’s fear of child’s own car accident. AOR: Adjusted odds ratios presented with 95% confidence intervals. “Child variable AOR” presents association adjusted by child-reported variables, and “all variable A OR” column presents association adjusted by both child- and parent-reported variables. All variables analyzed using multivariable logistic regression.

There was considerable overlap in significant socio demographic covariates between the two multivariable models, though prominent differences did arise. Maternal education was only found to be associated with fear of parental accident (p = 0.02), while maternal age and mother’s professional inactivity (with job seeking during unemployment considered inactive) both only associated with children’s fear of their own accident (both p<0.05).

### Fear of parental accident modelisation and car accidents death rate

**Fig 2:**
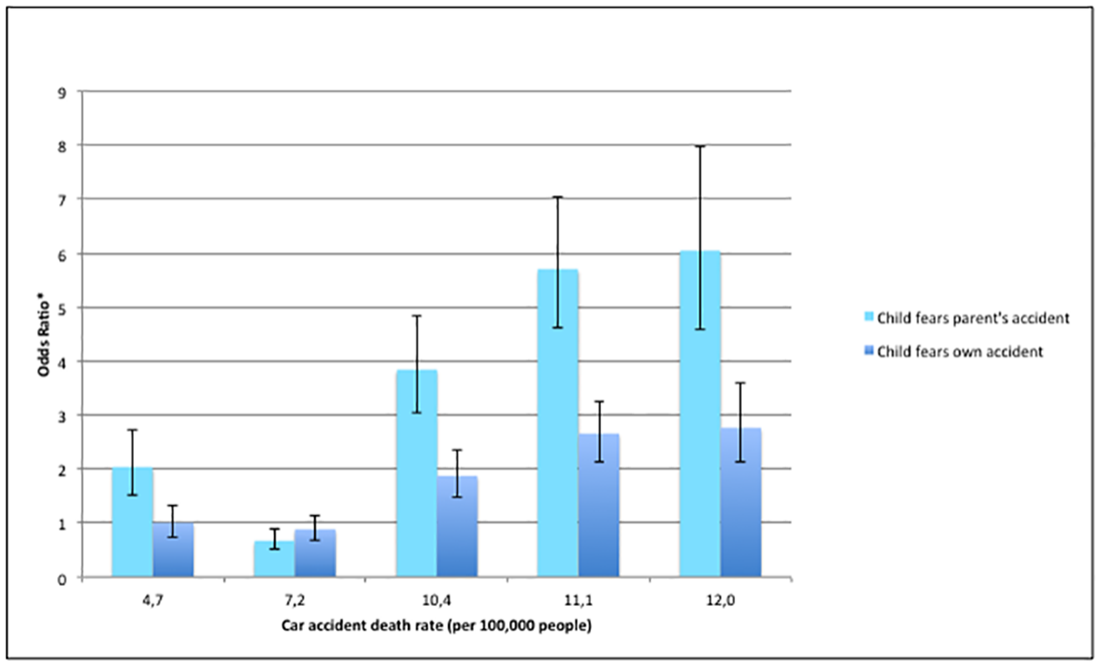
Odds of children’s fears are associated with car accident death rates (per 100,000 people) in an international sample.

The ecological variable, car accident death rate per 100,000 population, was highly significant and indicated that higher rates were associated with increased odds of reporting fear of a parent’s accident (p<0.0001). This suggested a dose-response effect (Fig 2).

Though this effect was largely consistent, those living in a country with a rate of 7.2 deaths (Italy) actually countered the trend and had lower odds of fear than the reference (OR = 0.646, p = 0.001). Additionally, those (Turkey) with the greatest death rate (12 per 100,000), had a slightly lower odds ratio for fear of parental accident than those (Lithuania, Romania) with the next highest (11.1 per 100,000).

As shown in Table 2, the odds of a child answering affirmatively to having a fear of a parent’s car accident were lower for each increasing year of age (OR = 0.911, 95% CI = 0.869, 0.955) and greater for female children than males (OR = 1.354, 95% CI = 1.191, 1.539). Because this item belongs to an assessment of Separation Anxiety Disorder, this is in line with expectations. Internalizing disorders are more frequent in females and for separation anxiety less likely with increasing age. Additionally, children were more likely to report fear if they lived with a single parent (OR = 1.258, 95% CI = 1.038, 1.525) or a mother with lower educational attainment (p = 0.023). Children of mothers who had completed high school and not continued their formal education had greater odds of this fear than children of mothers who continued beyond high school (OR = 1.198, 95% CI = 1.040, 1.380), a factor that was not significantly associated with fear of their own accident. There was no difference in odds for children whose mothers hadn’t completed high school and those who continued beyond, though the association approached significance (p = 0.064).

### Fear of child’s accident model and car accident death rate

Car accident death rate per 100,000 people was again associated with children’s self-reported fears and echoed the dose-response effect for higher death rates (Fig 2). The association with fear of child’s own accident was non-significant for countries with death rates of 4.7 or 7.2, but highly significant overall (p<0.0001). Though non-significant, it is notable that once again the data suggested that those from a country with a death rate of 7.2 per 100,000 had lower odds of reporting said fear (OR = 0.858, 95% CI = 0.683, 1.078).

As shown in Table 3, odds of children reporting fear of their own car accident were again found to decrease with age (OR = 0.855, 95% CI = 0.818, 0.894), to be greater for females (OR = 1.564, 95% CI = 1.390, 1.760), and greater for children from a single parent home (OR = 1.237, 95% CI = 1.036, 1.477). Unlike in the parental accident analysis, maternal education was not significant for predicting a fear of one’s own accident. Covariates uniquely significant to this model included maternal age (p = 0.014) and mother’s professional inactivity (OR = 1.217, 95% CI = 1.065, 1.389). Children with mothers 35–40 and those with mothers older than 40 had 0.898 and 0.789 times the odds of those with mothers younger than 35, representing an inverse relationship with mother’s increasing age.

## Discussion

Prevalence of fears of parent or own car accident is remarkably different across countries as expected from the literature review. Fear decreased with age and is higher in girls than boys. Single parenthood consistently increased the two types of fears, consistent with studies indicating that the increase number of divorce in Estonia as in Finland were a cause of children fears increase over a 10 years period[18].

The role of mother education on fears could be interpreted as a proxy for socio economic statute, which has been reported to be linked to child fear numbers and intensity. Inactivity is more difficult to interpret since its meaning may vary across countries when mother singlehood is both linked to lower socio economic statute and a more stressful situation as it was hypothesized in the Nordic study. Although socio familial factors have been rarely studied, as stressed by Melzer [19], their relationships with anxiety disorders have been largely established.

There is a robust association between car accident death rates by country and children’s elevated odds of self-reported fears of either parents’ or their own car accident. The monotonic relationship revealed by the multivariable parental accident model showed odds of fear-reporting that were up to 5 times that of the reference group, and remained the same even after adjustment for a range of socio-demographic factors that had been found to significantly vary between countries. This large effect size indicates a major difference between the range of nations and appears to be explained by the car accident death rate per 100,000 people (as detailed by WHO research).

As fear reporting is significantly associated with car accident fatality rates and those rates also significantly vary by country, it would appear that there is a concern that question regarding matters of economic and technological development as a means of mental health diagnosis will be uniformly applicable. Indeed in the same study, we have reported invariance for the separation anxiety and generalized anxiety DI subscales, mainly attributable to divergences of average scale score between countries; more specifically the question on parent car accident was invariant in the separation anxiety subscale while that on child own accident was not invariant for generalized anxiety [28].

Theoretically, fear reporting could have two different origins not mutually exclusive: as a rational response to a frequent dangerous event which in turn creates anxiety, or an irrational response to a threat that is incorrectly perceived as more dire and pressing than the reality. The first possibility would therefore not suggest psychopathology, whereas the latter situation indicates an anxious mental health issue [11, 29]. The current study’s results would seem to indicate the former is more plausible, with highly elevated odds of fear corresponding to high car accident death rates, and emphasizes the difficulty and hazard of using a diagnostic tool using such questions uniformly across a number of countries that exhibit very different cultural and economic histories. In the current study, because children in nations with higher death rates from car accidents seem to report car accident fears as, perhaps, a function of their environment, they may seem to have a correspondingly higher likelihood of being diagnosed with mental disorders than children exhibiting the same behavior but living in a low-accident (or high-income) country because an affirmative response to either fear question is a diagnostic factor in an anxiety disorder diagnosis.

Given the apparent variance in diagnostic value of the anxiety disorder questions depending on cultural context, it appears that cultural competency concerns are founded. Much cultural competency discourse has focused on better understanding of provision of care to racial minority populations, but illustrates the difficulty of assessing mental health burden across peoples with very different cultural histories and lived experiences [30, 31]. Oftentimes, there is not equivalence when diagnostic measures are taken from one context to the next due to differing connotations, idioms, definitions of symptoms and thresholds for what qualifies as illness. Transfer of metrics between cultures where there is cultural proximity between the languages (such as French and English) may be easier and more valid, as the concept behind idioms is fairly similar [32]. In this manner, car accidents may be a faulty measure of anxiety symptoms at least for cross-country comparisons but this may concern intra country comparisons as well, as fear of accidents varies with context.

An alternative explanation for these results is that another environmental circumstance tied to countries’ economic development may account for the trend that emerged upon car accident rate grouping. Among them the level of parental anxiety including anxiety linked to fear of car accidents. Indeed, the countries that exhibited the highest relative odds of fear reporting in both models were the four middle-income nations (Bulgaria, Lithuania, Romania, Turkey) rather than the high-income (Germany, Italy), as classified in the aforementioned WHO report [1]. Research has shown that in developing countries common mental disorders among them anxio- depressive disorders are associated with a number of poverty and socioeconomic-related factors [33]. Possible explanations for the association between low socioeconomic status and diminished mental health have been poor living conditions and housing, rapid and unpredictable social change and ensuing feelings of loneliness, hopelessness due to the shame of one’s social status, and poor education. Indeed, it was found that maternal education, a variable which often associates with children’s mental health issues, was found to vary across the 7 nations in the SCHME sample (Table 1).

As for maternal characteristics, many appear to be tied to development of fears that contribute to anxiety disorders and may be intervention opportunities [33]. Some of these factors are professionally-related and would appear to be the most amenable to change. For example, the association between mothers pursuing education beyond high school with fear of parental accident, as well as between mothers who are professionally inactive and fears of children’s own accidents, may be addressed by better professional development opportunities for mothers and an emphasis on continuing education. Previous studies have found that better maternal education leads to better health for children. This has been attributed to mothers’ greater general and specific knowledge, access to information and mothers’ improved agency to control their immediate circumstances [34]. This improved agency would explain why lower educational attainment and professional inactivity both were associated with accident fears, as either factor would limit mothers’ freedom to effect change on their environments in a way that would shield their children from causative exposures. Thus, the current study highlights the possibility of a maternally focused intervention that may yield several beneficial effects.

Given the fairly robust relationship between high death rate and high fear reporting, the uncharacteristically low fear reporting among children in Italy (children drawn from Sardinia), where the car accident death rate is among the highest at 7.2 per 100,000, was unusual. There are several possible explanations. For one, it is possible that Sardinia is an inaccurate representation of the state of children’s fears across the nation. Notably, Sardinia is an island and autonomous region that does not have the super highways (Autostrade) that are present in the rest of Italy. Outside of regional issues, there is the possibility that Italian children simply have a lower rate of reporting internalizing disorders in general. For example, a study by Kovess *et al*. [35] exploring children’s suicidal ideation in this sample found that Italian children reported the lowest prevalence of thoughts of suicide or death among all countries surveyed. Regardless of whether this low reporting is due to actual lower prevalence of disorders or bias, this atypical circumstance may explain why Italy bucks the current trend.

The current study must be received with some considerations. As the study is based on a cross-sectional survey, direct causality cannot be determined. Additionally, there is danger of ecological fallacy since death rates were applied uniformly so that every individual from a country was assigned the value for their country, despite the fact that on a smaller scale, individuals may live in regions of higher or lower rates. Further, the representativeness of each nation’s children for the entire population varies. For example, Lithuania’s survey was performed nationwide and is therefore nationally representative, whereas Italy’s, as mentioned previously, was conducted entirely in the Sardinia region and therefore may not be representative if the region differs greatly from the rest of the nation. In such a situation there is then a validity concern, in that what is being posited as a “cross-country” comparison is actually a comparison between the country of Lithuania and a region within Italy. Additional nuances of the sample were that Germany’s participants were all collected from rural areas, Romania and Turkey’s were largely urban, Bulgaria’s sample was almost equally half urban and half rural, and the Netherlands didn’t distinguish between rural or urban regions. The salient detail is that car accident death rates likely vary between rural or urban regions because of differing density of automobiles, walkability or availability of public transportation. If the assigned car accident death rates overestimated the actual exposure individuals in the sample experienced in their nearby environment, this would likely attenuate the observed effect of death rate on the prevalence of child’s fears. On the other hand, underestimation of death rate would indicate a high prevalence of fear despite low death rate and therefore exaggerate the effect of accident death rates on fear development. The car accident death rate indicator may be representative as assigned, however, as the theorized effect of accident death frequency is indirect, and therefore any region within a country with the nationally shared culture and language may experience the effects of the national death rate through the sharing of information among citizens regarding the frequency of car accident deaths. Encouragingly, the association between death rates and fear of accidents remained even after adjustment by multiple potential confounders, increasing the likelihood that the main effect of death rate on fear prevalence was being observed. Yet, because no sub-national indicator for the level of development or automobile density of different regions was collected, an assumption that each sample represents the country it came from is necessary. Further inquiry may be well served by including indicators for this aspect of participant’s environment. Finally they were some missing parental reports, however this missing data is unlikely to be related to the main exposure in question (car accidents in the surrounding environment) and therefore is not believed to greatly bias the results. We note that we accounted for within-country clustering by including countries as fixed effects in regression modeling. An alternative approach would be to consider countries as random effects. However, fixed effects modeling is an appropriate and valid strategy for a sample in which there are a small number (seven) clusters, and when considering only one country-level covariate (car accident death rate). Thus, there is little concern for bias in the estimation of the standard errors given the fixed effects design, compared with a random effects approach.

## Conclusions

The current study indicates that some indicators that are closely tied to the technological/development context within which children live may not be good indicators of mental health conditions in cross cultural comparisons, since measuring instruments and the diagnostic factors of interest may have unequal diagnostic power across different populations.

The mechanism driving the relationship between car accident fatalities and accident fear reporting is unclear, but seems to not be related to whether children have directly experienced an injury or accident in the past year. As children seem to be indirectly affected by the frequency of car accident deaths in their countries, this presents opportunities for intervention at an upstream point in the causal pathway. Broadly speaking, this may be evidence of the importance of infrastructural development—namely, bettering roads and driving conditions. It will be important for nations to be aware of these effects so that proper endeavors can be taken to keep roadways safe, sensitize parents to the psychological effects their driving can have on their children, and address the increased need for mental health care in the face of development.

Ultimately the link between child fears and car accident fatalities may be used in safety campaigns to sensitize parents to their driving attitudes.

## Supporting information

S1 File (XLS)
